# Ageing people with intellectual disabilities and the association between frailty factors and social care: A Swedish national register study

**DOI:** 10.1177/17446295211037170

**Published:** 2021-11-02

**Authors:** Gerd Ahlström, Eva Flygare Wallén, Magnus Tideman, Marianne Holmgren

**Affiliations:** Lund University, Sweden; Karolinska Institutet (KI), Sweden; Municipality of Östersund, Sweden; Halmstad University, Sweden; Ersta Sköndal Bräcke University, Sweden; Lund University, Sweden

**Keywords:** frailty, intellectual disability, multimorbidity, older people, social care

## Abstract

The aim of this study was to describe the social care provided for different age groups of people with intellectual disability, 55 years or above, and to investigate the association between such care and frailty factors for those with diagnosed level of intellectual disabilities. Descriptive and logistic regression analyses were used. Commonest forms of social care among the 7936 people were *Residential care*, *Daily activities* and *Contact person*. *Home help* and *Security alarm* increased with age. The frailty factors significantly associated with increased social care were age, polypharmacy and severe levels of intellectual disabilities. Persons most likely to be in residential care were in the age group 65–79 with polypharmacy and severe disability. The results indicate a need for further research of how frailty factors are considered in social care and longstanding medication, especially then severe intellectual disability hinders communication. A national strategic plan for preventive interventions should be developed to ensure the best possible healthy ageing.

## Introduction

Longevity has increased in people with intellectual disability during the last three decades (Coppus, 2013; Dieckmann et al., 2015; Ng et al., 2017), which adds increasing age-related frailty to the disability and health problems experienced by people with intellectual disabilities from their early years (Evenhuis et al., 2012; Schoufour et al., 2013). Despite the fact that multimorbidity is highly prevalent in ageing people with intellectual disabilities (Hermans and Evenhuis, 2014; McCarron et al., 2013), a Swedish national population study showed decreasing of specialist healthcare with age. The oldest age group (85–94 years) had less than the younger age groups (45–84 years) and less than the control group without intellectual disabilities in the same age-range (Sandberg et al., 2016), which is unexpected from an ageing perspective. This result contrasts with the existing evidence concerning the general population, which indicates greater healthcare utilisation with more advanced age (Akugizibwe et al., 2020; Doheny et al., 2019; Lehnert et al., 2011). Despite the insufficiency of generally accepted measures for frailty in the literature, specialist health care or hospital care may be seen as one adverse health outcome in relation to older age, as studied in the general population (Akugizibwe et al., 2020; Doheny et al., 2019; Lehnert et al., 2011), but has not been in focus as a frailty factor for people with intellectual disabilities. Valid register-based data on specialist health care has the advantage of not being dependent on self-report, which has weaknesses when it comes to people with intellectual disabilities because of limited understanding and communication problems (Schoufour et al., 2013). Multimorbidity and repeated healthcare visits constitute a risk of polypharmacy in older people, leading to side effects from inappropriate medications (Axmon et al., 2017). In an Irish national sample of 753 persons with intellectual disabilities, polypharmacy was strongly associated with living in a residential institution compared with independent living or living in a group home, but there was no significant relationship between polypharmacy and age or gender (O’Dwyer et al., 2016). Moreover, the severity level of intellectual disability is a well-known significant frailty factor, and those with a moderate, severe or profound level are more often living in residential care for people with intellectual disabilities and have a larger need of service and support for daily activities than those with a mild level (El Mrayyan, 2020; Schoufour et al., 2013). Living longer has consequences for age-related morbidity, with implications for health monitoring and preventive interventions by staff providing social care for healthy ageing (Haveman et al., 2010; Marks and Heller, 2003; World Health Organization, 2001).

Until 50 years ago, people with intellectual disabilities in Sweden lived in large institutions governed from a medical perspective (Ericsson, 2005; Tossebro et al., 2012). Then the Swedish Government resolved that these institutions should be closed and that the implementation of the policy should be handed over to the local governments (290 municipalities). The deinstitutionalisation process started in 1970 and was completed in 2003. The early part of this period was characterised by an intensive ideological debate about normalisation, empowerment, integration and autonomy (Ericsson, 2005; Tossebro et al., 2012), and there was an introduction of a new rights-based legislation regarding municipally based social care for people with severe disabilities (SFS 2020:441).

The improved living conditions resulting from individualised support to live like other citizens in the community after deinstitutionalisation has led to an increased number of older people with intellectual disabilities (McCarron et al., 2019; World Health Organization, 2001). Responding to the needs of ageing people with intellectual disabilities is a complicated challenge and there are gaps where neither the disability service nor elderly care can adequately meet these needs (Bigby, 2002). Staff in elderly care facilities have limited experience and are not trained to meet the particular needs of older people with intellectual disabilities, who commonly live at a great geographical distance from each other. Staff in the disability service are similarly inexperienced with regard to the ageing process, which can even result in age discrimination (World Health Organization, 2015). Furthermore, negative attitudes on the part of decision-makers and staff in the disability field may create barriers to accessing the available social care services (Bigby, 2002). It is important, especially in the light of the increase in longevity, to explore what community-based social care services exist for older people with intellectual disabilities, and to explore whether they are adapted to these people’s unique needs. Therefore, the aim of this national study was to describe the social care services provided to different age groups of people with intellectual disabilities, 55 years or above. A further aim was to investigate the association between received social care and the frailty factors *Age*, *Multimorbidity*, *Specialist healthcare*, *Polypharmacy* and *Level of intellectual disability* for persons with a diagnosed level of intellectual disability. We apply the World Health Organization (WHO) definition of frailty as a ‘clinically recognisable state in which the ability of older people to cope with everyday or acute stressors is compromised by an increased vulnerability brought by age-associated declines in physiological reserve and function across multiple organ systems’ (Hoogendijk et al., 2019; World Health Organization, 2016: viii).

## Methods

This study uses longitudinal data from four Swedish national registers both to identify the study cohort (the Register for social care and services decided through the Swedish Act Concerning Support and Service for People with Certain Functional Impairments [abbreviated as LSS register]) and for outcomes variables (the LSS register, the Social Service Register [SoL], the National Patient Register [NPR] and the Swedish National Register of Prescribed Drugs).

### Context and disability policy

Swedish disability policy is based on human rights and thus on a humanistic view which stresses all people’s equal value regardless of their ability to function (SFS 2020:441; United Nations, 2006). People with comprehensive and longstanding disabilities are entitled to specific social care services to manage their daily lives. The basic legislation for fulfilling this entitlement is the Act Concerning Support and Service for Persons with Certain Functional Impairments, abbreviated in Swedish as LSS (SFS 2020:441). The law applies to three separate groups, one of which is people with intellectual disabilities, autism or autism spectrum disorder (SFS 2020:441). In 2019, this group comprised 89% of all who were provided with social care services as mandated by the LSS (The National Board of Health and Welfare, 2020). The LSS is a strong rights law and does not imply any restriction of rights deriving from other laws. Alternative or complementary support can be received under the Social Services Act (abbreviated in Swedish, as SoL), which applies to all Swedish citizens (SFS 2020:1260). Decisions on LSS and SoL social care services are always preceded by an investigation of the person’s needs, performed by a social worker. The social care services are mainly financed through taxes, and the municipalities are responsible for providing both LSS and SoL services.

### Sampling of study cohort

The national study cohort was constructed by identifying all people with intellectual disabilities, autism or autism spectrum disorder, as recorded in the LSS register. In this study, all three were classified as intellectual disabilities. The inclusion criteria were the following: receiving at least one LSS service, aged 55 or older and alive at the end of 2012. The choice of 55 or older as an inclusion criterion for ageing is based on previous research recognising that people with intellectual disabilities tend to age earlier than the general population (Coppus, 2013). The number of people in the study group was 7936, composed of 4327 men (54.5%) and 3609 women (45.5%). Their mean age in 2012 was 64.2 years.

### Outcome variables selected from registers

The National Board of Health and Welfare, an authority under the control of the Swedish government, extracted available outcome data retrospectively for the study period 2002–2012 on the included individuals from the four national registers: the LSS register (available from 2004), the SoL register (available from 2007), the NPR (complete coverage since 1987) and the Swedish National Register of Prescribed Drugs (available from 2006). Generalisability of the data is high due to mandatory registration and continuous quality assurance of data by the responsible authority (Ludvigsson et al., 2011; The National Board of Health and Welfare, 2020).

Data selected from the LSS register were age, sex and the eight social care services for adults for the study period 2004–2012, namely, *Companion service*, *Contact person*, *Daily activities, Intellectual disability (ID) residential care*, *Personal assistance*, *Relief service*, *Short stay away from home*, and *Specially adapted housing* (see definitions in Table 1).

**Table 1.** Description of social care and services in accordance with the LSS and complementary SoL social care and services for older people with intellectual disabilities.

	**Short description of the social care and services**
**LSS social care and services**	
Companion service	A person who is not entitled to the service Personal assistance (see below) may instead be entitled to companion service. A companion makes it easier for people with extensive disabilities to participate in community life, for example to visit friends, participate in leisure activities or cultural life.
Contact person	Personal support, to break social isolation and to facilitate an independent life. Helping the person participate in recreational activities and providing advice in everyday situations.
Daily activities	Meaningful activities for people of working age who lack gainful employment and are not studying.
ID residential care (includes different housing with varied level of assistance, service and care)	Group accommodation and service housing are the most common forms. Group accommodation consists of three to five separate flats connected in one building. The person is provided with assistance, service and care by staff 24 hours a day according to their needs. Service housing is available for persons who are more independent. It consists of separate flats spread out over a larger area, and the staff can be available up to 24 hours a day.
Personal assistance	Persons with major functional impairment and extensive need for support and help in their everyday lives are entitled to personal assistance for a maximum of 20 hours a week.
Relief service	Means of enabling relatives (informal carers) to relax or do chores outside the home. Can be offered both as a regular intervention and as a solution for unexpected situations.
Short stay away from home	Means of enabling a person with a disability to experience a change of environment and recreation and to provide relief for carers. The stay can be located in a short-term home, with a support family or at a holiday camp.
Specially adapted housing	Housing with certain basic adaptations but without permanent staffing, designated by the municipality. This form of housing does not include nursing, leisure activities or cultural activities.
**SoL social care and services**	
Companion service	Same goal as in LSS (see above) but for persons not fulfilling the LSS criteria. This service can be a part of home help or living support.
Contact person/family	Person or family with the task of supporting and helping the person with intellectual disability and their next of kin in personal matters.
Day activities	Daytime activities for older and people with disabilities in need of stimulation and rehabilitation. Primarily target those with dementia or mental disabilities. Daytime activities help many to continue to live in their homes.
Elderly residential care	Special types of housing for service and care around 24 hours a day. For older persons who, for physical, mental or other reasons, have significant difficulties in daily life and need such accommodation.
Home help (Personal ADL, cleaning and food distribution)	Service in the person’s home, such as personal activities of daily living, and/or personal care such as cleaning or food distribution. Other practical help such as laundry, shopping, post and bank errands, and help with meal preparation.
Living support	Support in daily living for specific target groups (people with psychiatric, neuropsychiatric or cognitive disability), consisting of support in the home, in social life and in contact with authorities and healthcare institutions.
Relief service	Means of enabling next of kin (informal caregivers) to relax or do chores outside the home (part of a home help service decision).
Security alarm	Assistance with security alarms, commonly to be worn on the arm and/or installed in the bed or bathroom.
Short stay away from home	Assistance in the form of temporary housing for relief and a change of environment. Same goal as in LSS (see above) but for persons not fulfilling the LSS criteria.

ID residential care = residential care for people with intellectual disabilities; LSS = *Lagen om stöd och service till vissa funktionshindrade* (the Swedish Act Concerning Support and Service for People with Certain Functional Impairments); SoL = *Socialtjänstlagen* (the Swedish Social Services Act).

The services included from the SoL register for the study period 2007–2012 (excluded 2009) were Companion service, Contact person/family, Day activities, Elderly residential care, Home help, Living support, Relief service, Security alarm, Short stay away (see definitions in Table 1). In accordance with the recommendation by the National Board of Health and Welfare, data from 2009 were excluded because the data quality was unsatisfactory.

Three of the frailty factors in this study were extracted from the NPR register for the study period 2002–2012: *Specialist healthcare* (covering all inpatient and outpatient specialist healthcare visits for each person in the study group), *Multimorbidity* and *Level of intellectual disability*. The diagnoses in the NPR for multimorbidity and level of intellectual disability are coded according to the International Statistical Classification of Diseases and Related Health Problems, 10th Revision (ICD-10). For inpatient care, one primary diagnosis (main cause) and up to 21 secondary diagnoses (contributory causes) are recorded; for outpatient care, one primary diagnosis and 15 secondary diagnoses are recorded. The diagnosis is registered at discharge, which means that it is based on careful examination by the medically responsible physician. Data on level of intellectual disability were available for 1151 persons (14.5%) in the study group due to registering only when it is the cause of a specialist care visit.

The frailty factor *Polypharmacy* was extracted for the study group from the Drug Prescription Register for the study period 2006–2012, which contains data on all purchases of prescribed drugs in Sweden. The purchases are registered at the date of dispensation by all pharmacies, and the drug is reported according to the Anatomical Therapeutic Chemical (ATC) classification system. For this outcome, specific drugs that were assessed as being of importance for common sicknesses among older people with and without intellectual disabilities were selected. Several of these involve a high risk of side effects, especially for people with intellectual disabilities. The drugs fell into the following ATC categories: Alimentary tract and metabolism (A02BC, A06A, A10A, A10B, A12A), Blood and blood-forming organs (B01A, B01AC, B03A, B03B), Cardiovascular system (C01AA, C01D, C03, C07, C08, C09, C10AA), Genitourinary system and sex hormones (G04BD, G04C), Systematic hormonal preparation, excluding sex hormones and insulin (H03AA), Musculoskeletal system (M01A, M05B), Nervous system (N02A, N02B, N02C, N03A, N04B, N05A, N05B, N05C, N06A, N06BA, N06D), Respiratory system (R03) and Sensory organs (S01E).

### Data analysis

Descriptive statistics are presented as numbers and percentages. For those persons with a diagnosed level of intellectual disability, the association between frailty factors (*Age*, *Specialist healthcare*, *Multimorbidity*, *Polypharmacy* and *Level of Intellectual Disability*) and the four most common LSS services provided in 2012 (*Companion service*, *Contact person*, *Intellectual Disability residential care* and *Daily activities*) was assessed by multivariable logistic regression analysis (Bland, 2015; Peduzzi et al., 1996). The results were presented as OR and a 95% confidence interval. SoL services were excluded from the regression analysis due to the shorter available data period (2007–2012, with the exclusion of 2009).

For the frailty factor *Age*, the study group was divided into three age groups: 55–64 years, 65–79 years and 80 years or older. The first cut-off was at the mean age of the study group (64.2 years) and the second cut-off was the age at which people in general need more care and services (80 years and older). These age groups are also commonly used in the literature on ageing (Chang et al., 2019; World Health Organization, 2015). The cut-off for *Specialist healthcare* in the regression analysis was the median number of health care visits (inpatient and outpatient) in the study group, and the variable was dichotomised into low healthcare utilisation (<6 visits) and high healthcare utilisation (≥6 visits). *Multimorbidity* (Johnston et al., 2019) was calculated as the number of unique diagnoses for each person in the course of at least 1 year, dichotomised into no multimorbidity (<2 diagnoses) and multimorbidity (≥2 diagnoses); this cut-off is according to the definition in a systematic review (Kinnear et al., 2018). Both primary and secondary diagnoses were included but not the diagnoses for intellectual disabilities (F7, F8, F9 and Q90), then they were part of the inclusion criteria. *Level of Intellectual Disability* was identified as having at least one subdiagnosis of mild, moderate, severe or profound intellectual disabilities (F70, F71, F72 or F73) and dichotomised into mild level and moderate/severe/profound level. *Polypharmacy* was calculated as the number of selected drugs prescribed for at least 1 year during the period 2006–2012, dichotomised into no polypharmacy (<5 drugs) or polypharmacy (≥5 drugs), according to the commonly used definition (O’Dwyer et al., 2016, 2018).

Adjustment for sex was made in all models. IBM SPSS Statistics for Windows v.22 (IBM Corporation, Armonk, NY, USA) was used to perform the statistical analyses. A p-value below 0.05 was considered significant.

### Ethics

The study was approved by the Regional Ethical Review Board in Lund (approval no. 2013/15). In addition, the National Board of Health and Welfare performed separate secrecy review before providing access to an anonymised dataset 2015. It was not possible to ask each individual for consent to participate in the study, due to the requirement of anonymised data for the researchers. Instead, active refusal of participation was applied. This was done by publishing information about the planned study in the national newspaper *Dagens Nyheter* and in *UNIK*, the magazine of the National Association for People with Intellectual Disability (FUB), which has a circulation of 22,000. The target audience for *UNIK* is mainly FUB members (people with intellectual disabilities) and their families. The information was published in two versions, whereof one was easy-to-read text. Besides providing facts about the study, it provided guidance concerning how to contact the research manager by phone, email or mail in order to opt out of the study. The research manager was then responsible for contacting the national registrars so that those who refused participation were excluded from any data to be provided. The study was performed in accordance with the revised Declaration of Helsinki (World Medical, 2013).

## Results

More than half of the study group in 2012 were in the youngest age group, 55–64 years (58.7%, n = 4656), of whom 56.3% were men (n = 2620) and 43.7% women (n = 2036). More than one third were aged 65–79 years (37.2%, n = 2956), of whom 53.0% were men (n = 1568) and 47.0% women (n = 1388). The smallest group were those aged 80 years or older (4.1%, n = 324), of whom 42.9% were men (n = 139) and 57.1% women (n =185).

### Social care services and age group

Table 2 shows the number of persons receiving LSS services in 2012, the distribution for the three age groups, the number of those who received these services every year and the number of those who received the services in at least 1 year of the study period. The most common LSS services received were *Intellectual Disability residential care*, *Daily activities* and *Contact person*. Contact persons were commonly provided for people with intellectual disabilities who were 65–79 years of age but decreased in the oldest age group. The provision of *Intellectual Disability residential care* increased with higher age whilst *Daily activities* decreased with increasing age as a natural consequence of retirement. *Companion service* provided every year was not as frequent as *Contact person*, and 16 percent received it for at least 1 year during the study period 2004–2012 (Table 2).

**Table 2.** Number of older people with intellectual disabilities who received different forms of LSS social care and services in 2012, divided into age groups, and the number who received such services every year and at least 1 year during the whole study period 2004–2012.

2012					2004–2012	
LSS social care and services	Total study group (n = 7936)	Age group 55–64 years (n = 4656)	Age group 65–79 years (n = 2956)	Age group ≥80 years (n = 324)	Received every year (n = 7936)	Received in at least 1 year (n = 7936)
	n (%)	n (%)	n (%)	n (%)	n (%)	n (%)
Companion service	533 (6.7)	302 (6.5)	214 (7.2)	17 (5.2)	184 (2.3)	1279 (16.1)
Contact person	3524 (44.4)	2000 (43.0)	1395 (47.2)	129 (39.8)	1892 (23.8)	4895 (61.7)
Daily activities	4571 (57.6)	3599 (77.3)	945 (32.0)	27 (8.3)	3626 (45.7)	6354 (80.1)
ID residential care	6014 (75.8)	3352 (72.0)	2391 (80.9)	271 (83.6)	4759 (60.0)	6273 (79.0)
Personal assistance	132 (1.7)	82 (1.8)	50 (1.8)	0 (0)	68 (0.9)	285 (3.6)
Relief service	10 (0.1)	7 (0.1)	2 (0.1)	1 (0.3)	1 (≤0.1)	37 (0.5)
Short stay away from home	41 (0.5)	34 (0.7)	7 (0.2)	0 (0)	17 (0.2)	171 (2.2)
Specially adapted housing	53 (0.7)	34 (0.7)	19 (0.6)	0 (0)	2 (≤0.1)	292 (3.7)

*Note:* A person can have more than one form of social care service.

ID residential care = residential care for people with intellectual disabilities; LSS = *Lagen om stöd och service till vissa funktionshindrade* (the Swedish Act Concerning Support and Service for People with Certain Functional Impairments).

As shown in Table 3, receiving two LSS services was most common for the individuals in the whole group of older people with intellectual disabilities, and the median number of services was also two (not shown). The provision of LSS services varied between the age groups, and it was most common for those who were 80 years and older to receive only one service.

**Table 3.** Number and percentage of older people with intellectual disabilities who received one or more forms of LSS social care and services in 2012, divided into age groups.

Number of LSS social care	Total study group ≥ 55 years (n = 7936)	Age group 55–64 years (n = 4656)	Age group 65–79 years (n = 2956)	Age group ≥80 years (n = 324)
and services	n (%)	n (%)	n (%)	n (%)
One	2812 (35.4)	1288 (27.7)	1310 (44.3)	214 (66.0)
Two	3364 (42.4)	2027 (43.5)	1238 (41.9)	99 (30.5)
Three	1705 (21.5)	1299 (27.9)	395 (13.4)	11 (3.4)
Four	53 (0.7)	40 (0.9)	13 (0.4)	0 (0)
Five	1 (0)	1 (0)	0 (0)	0 (0)
Six	1 (0)	1 (0)	0 (0)	0 (0)

LSS = *Lagen om stöd och service till vissa funktionshindrade* (the Swedish Act Concerning Support and Service for People with Certain Functional Impairments).

The most common social care services provided in accordance with the SoL in the total study group were *Home help* and *Security alarm* (Table 4). However, in the youngest age group (55–64 years) *Living support* was more common than *Security alarm*. Receiving *Home help*, *Security alarm* and *Elderly residential care* increased from 65 years.

**Table 4.** Number of older people with intellectual disabilities who received various forms of SoL social care and services in 2012, divided into age groups, and the number who received such services every year and at least 1 year during the study period 2007–2012 (not 2009^†^).

2012					2007–2012 (excluding 2009)^†^	
SoL service	Total study group n = 1916	Age group 55–64 years n = 908	Age group 65–79 years n = 802	Age group ≥80 years n = 206	Received every year during the study period n = 2710	Received at least 1 year during the study period n = 2710
	n (%)	n (%)	n (%)	n (%)	n (%)	n (%)
Companion service	236 (12.3)	79 (8.7)	127 (15.8)	30 (14.6)	19 (0.7)	281 (10.4)
Contact person	68 (3.5)	47 (5.2)	20 (2.5)	1 (0.5)	3 (0.1)	86 (3.2)
Day activities	135 (7.0)	43 (4.7)	71 (8.9)	21 (10.2)	21 (0.8)	194 (7.2)
Elderly residential care	405 (21.1)	108 (11.9)	210 (26.2)	87 (42.2)	75 (2.8)	544 (20.1)
Home help	1240 (64.7)	531 (58.5)	550 (68.6)	159 (77.2)	295 (10.9)	1276 (47.1)
Living support	509 (26.6)	382 (42.1)	121 (15.1)	6 (2.9)	146 (5.4)	619 (22.8)
Relief service	23 (1.2)	9 (1.0)	10 (1.2)	4 (1.9)	2 (0.07)^‡^	28 (1.0)
Security alarm	792 (41.3)	279 (30.7)	382 (47.6)	131 (63.6)	238 (8.8)	940 (34.7)
Short stay away	95 (5.0)	25 (2.8)	51 (6.4)	19 (19.2)	2 (0.07)^§^	128 (4.7)

*Note:* A person can have more than one form of social care and services.

^†^ In accordance with the recommendation by the National Board of Health and Welfare, data from 2009 were excluded because the data quality was unsatisfactory.

^‡^Maximum 3 years.

^§^Maximum 4 years.

SoL = *Socialtjänstlagen* (the Swedish Social Services Act).

### Frailty factors

The distribution of frailty factors in older persons with intellectual disabilities who received or did not receive the four most commonly provided LSS social care services in 2012 is shown in Table 5. For *Age*, those aged 65 years and older received in percent less services than those in the youngest age group but 83.6% (271 of 324 persons) in the age group 80 years or older lived in *Intellectual Disability residential care*. About half of those with six or more *Specialist healthcare* visits during the 11-year study period received social care and services 2012. Also, those with *Multimorbidity* (≥2 diseases besides intellectual disability) received a high level of service. Everyone in the study group had been prescribed at least one drug, but *Polypharmacy* (≥5 drugs) was more common among those who lived in *Intellectual Disability residential care*. Concerning *Level of Intellectual Disability*, *Companion service* and *Contact person* were more common among those with mild intellectual disabilities, whilst *Intellectual Disability residential care* and *Daily activities* showed similar percentages for persons with mild intellectual disabilities and moderate/severe/ profound intellectual disabilities (Table 5).

**Table 5.** Distribution of frailty factors in older persons with intellectual disabilities who received, and in those who did not receive, the four most common forms of LSS social care and services in 2012.

	Total study	Companion service		Contact person		ID residential care		Daily activities	
Frailty factors	group	Yes	No	Yes	No	Yes	No	Yes	No
2002–2012	n (%)	n (%)	n (%)	n (%)	n (%)	n (%)	n (%)	n (%)	n (%)
**Age in 2012**	**7936 (100.0)^†^**	**n = 533**	**n = 7403**	**n = 3523**	**n = 4413**	**n = 6014**	**n = 1922**	**n = 4571**	**n = 3365**
55–64	4656 (58.7)	302 (56.7)	4354 (58.8)	2000 (56.8)	2656 (60.2)	3352 (55.7)	1304 (67.8)	3599 (78.7)	1057 (31.4)
65–79	2956 (37.2)	214 (40.2)	2742 (37.0)	1394 (39.6)	1562 (35.4)	2391 (39.8)	565 (29.4)	945 (20.7)	2011 (59.8)
≥80	324 (4.1)	17 (3.2)	307 (4.1)	129 (3.7)	195 (4.4)	271 (4.5)	53 (2.8)	27 (0.6)	297 (8.8)
**Specialist healthcare 2002–2012**	**6518 (82.1)^†^**	**n = 442**	**n = 6076**	**n = 2884**	**n = 3634**	**n = 4995**	**n = 1523**	**n = 3647**	**n = 2871**
<6 (low utilisation)	3242 (49.7)	202 (45.7)	3040 (50.0)	1406 (48.8)	1836 (50.5)	2533 (50.7)	709 (46.6)	1868 (51.2)	1374 (47.9)
≥6 (high utilisation)	3276 (50.3)	240 (54.3)	3036 (50.0)	1478 (51.2)	1798 (49.5)	2462 (49.3)	814 (53.4)	1779 (48.8)	1497 (52.1)
**Multimorbidity 2002–2012**	**6405 (80.7)^†^**	**n = 438**	**n = 5967**	**n = 2835**	**n = 3570**	**n = 4903**	**n = 1502**	**n = 3576**	**n = 2829**
<2 (no multimorbidity)	963 (15.0)	59 (13.5)	904 (15.1)	438 (15.4)	525 (14.7)	732 (14.9)	231 (15.4)	588 (16.4)	375 (13.3)
≥2 (multimorbidity)	5442 (85.0)	379 (86.5)	5063 (84.9)	2397 (84.6)	3045 (85.3)	4171 (85.1)	1271 (84.6)	2988 (83.6)	2454 (86.7)
**Polypharmacy 2006–2012**	**7936 (100.0)^†^**	**n = 533**	**n = 7403**	**n = 3523**	**n = 4413**	**n = 6014**	**n = 1922**	**n = 4571**	**n = 3365**
<5 (no polypharmacy)	2839 (35.8)	206 (38.6)	2633 (35.6)	1345 (38.2)	1494 (33.9)	1925 (32.0)	914 (47.6)	1844 (40.3)	995 (29.6)
≥5 (polypharmacy)	5097 (64.2)	327 (61.4)	4770 (64.4)	2178 (61.8)	2919 (66.1)	4089 (68.0)	1008 (52.4)	2727 (59.7)	2370 (70.4)
**Level of ID 2002–2012**	**1151 (14.5)^†^**	**n = 61**	**n = 1090**	**n = 489**	**n = 662**	**n = 930**	**n = 221**	**n = 676**	**n = 475**
Mild	611 (53.1)	39 (63.9)	572 (52.5)	296 (60.5)	315 (47.6)	453 (48.7)	158 (71.5)	332 (49.1)	279 (58.7)
Moderate/Severe/Profound	540 (46.9)	22 (36.1)	518 (47.5)	193 (39.5)	347 (52.4)	477 (51.3)	63 (28.5)	244 (50.9)	196 (41.3)

*Note:* A person can have more than one form of social care and services.

ID residential care = residential care for people with intellectual disabilities; Level of ID = Level of intellectual disability.

^†^ Percentage of total number, 7936.

LSS = *Lagen om stöd och service till vissa funktionshindrade* (the Swedish Act Concerning Support and Service for People with Certain Functional Impairments).

Regression analyses were performed on the persons with a diagnosed level of intellectual disability to assess the association of frailty factors with the likelihood that persons with intellectual disabilities received LSS services in 2012. As shown in Table 6, *Intellectual Disability residential care* was more likely to be provided in the *Age* group 65–79 years (OR 1.83), those with *Polypharmacy* (OR 2.74) and those with higher (moderate/severe/profound) *Level of Intellectual Disability* (OR 2.44). *Daily activities* were also more likely to be provided to those with moderate/severe/profound *Level of Intellectual Disability* than those with mild intellectual disabilities (OR 1.40), but less likely for those in the higher *Age* groups (65–79 years, OR 0.12; ≥80 years, OR 0.026) than for the youngest age group. Furthermore, those with *Polypharmacy* were less likely to have the LSS *Companion service* (OR 0.32) than those without polypharmacy. Those with a moderate/severe/profound *Level of Intellectual Disability* were less likely to have a *Contact person* (OR 0.61) than those with mild intellectual disabilities.

**Table 6.** Logistic regression between frailty factors and the four most common forms of LSS social care and services in 2012 among older persons with intellectual disabilities.

	Companion service	Contact person	ID residential care	Daily activities
Frailty factors	Yes (n = 60) vs No (n = 1035)	Yes (n = 469) vs No (n = 626)	Yes (n = 880) vs No (n = 215)	Yes (n = 640) vs No (n = 455)
2002–2012	Odds ratio (95% CI)	Odds ratio (95% CI)	Odds ratio (95% CI)	Odds ratio (95% CI)
**Age in 2012**				
55–64	Ref.	Ref.	Ref.	Ref.
65–79	0.78 (0.44–1.39)	1.10 (0.85–1.42)	1.83 (1.29–2.59)	0.12 (0.088–0.16)
≥80	0.61 (0.080–4.69)	0.81 (0.37–1.80)	1.24 (0.48–3.20)	0.026 (0.0060–0.11)
**Specialist healthcare 2002–2012**				
<6 (low utilisation)	Ref.	Ref.	Ref.	Ref.
≥6 (high utilisation)	1.82 (0.88–3.76)	1.21 (0.88–1.65)	0.86 (0.58–1.28)	0.96 (0.67–1.38)
**Multimorbidity 2002–2012**				
<2 (no multimorbidity)	Ref.	Ref.	Ref.	Ref.
≥2 (multimorbidity)	0.77 (0.31–1.93)	0.91 (0.58–1.42)	0.75 (0.42–1.35)	0.81 (0.48–1.36)
**Polypharmacy 2006–2012**				
<5 (no polypharmacy)	Ref.	Ref.	Ref.	Ref.
≥5 (polypharmacy)	0.32 (0.17–0.60)	0.99 (0.68–1.42)	2.74 (1.82–4.11)	0.91 (0.59–1.40)
**Level of ID 2002–2012**				
Mild	Ref.	Ref.	Ref.	Ref.
Moderate/Severe/Profound	0.72 (0.41–1.24)	0.61 (0.47–0.78)	2.44 (1.75–3.40)	1.40 (1.05–1.86)
**Sex**				
Female	Ref.	Ref.	Ref.	Ref.
Male	0.85 (0.50–1.45)	0.93 (0.73–1.18)	1.01 (0.74–1.39)	1.12 (0.84–1.48)

Bold indicates statistically significant results; CI = confidence interval.

ID residential care = residential care for people with intellectual disabilities; Level of ID = Level of intellectual disability; LSS = *Lagen om stöd och service till vissa funktionshindrade* (the Swedish Act Concerning Support and Service for People with Certain Functional Impairments).

## Discussion

The main results show that *Intellectual Disability residential care*, *Daily activities* and *Contact person* were the most common LSS social care and services in all age groups while *Contact person* was least common in the oldest age group. The most common complementary social care services received in accordance with the SoL were *Home help* and *Security alarm*. The provision of the LSS service *Intellectual Disability residential care* and the SoL services *Elderly residential care*, *Home help* and *Security alarm* increased with age. On the other hand, those aged 80 years and older received on average only one LSS service, compared with two services in the case of the younger age groups. Our study group consisted of people who met the requirements for receiving social care services in accordance with the LSS; therefore, it is not surprising that LSS services were more common than the ones provided in accordance with the SoL, which are complementary.

The frailty factors with significant association to increased social care and services were age, polypharmacy and severe level of intellectual disability. Those most likely to live in *Intellectual Disability residential care* were people in the age group 65–79 years who had polypharmacy and more severe levels of intellectual disabilities. However, *Contact person* was more likely among those with mild intellectual disabilities than among those more frail, i.e. with severe intellectual disabilities, which is unsurprising in that those with mild intellectual disabilities are not expected to need access to staff around the clock. Our results confirm those of previous studies in respect of the high proportion of polypharmacy among people with intellectual disabilities (Axmon et al., 2017; O’Dwyer et al., 2016) and the greater likely exposure to polypharmacy of persons in residential care and with severe/profound intellectual disabilities (O’Dwyer et al., 2016). Furthermore, half of the people were prescribed at least one potentially inappropriate medication (PIM) during a year, as compared to 9% in the group from the general population (Axmon et al., 2017). The research indicates the importance of access to medical competence for people with intellectual disabilities in a residential care setting. However, managers reported that in the recruitment of staff they focused on personal values and qualities rather than on specific experience, training and qualifications (Northway et al., 2017). Thus there is a need for training and increased knowledge to enable care staff to monitor signs of adverse side effects of drugs. In addition there is a need to build up close collaboration with a geriatrician or GP for repeated reviews of ongoing medication to facilitate change of treatment when side effects are identified.

Previous research has shown a high rate of occurrence of multimorbidity among older people with intellectual disabilities, from 72–80% among those 50 years or older (Hermans and Evenhuis, 2014; McCarron et al., 2013). It was somewhat higher in the present study, 85% among those above 55 years. Specialist healthcare or hospital care has been included as frailty factor in research on older people in the general population (Akugizibwe et al., 2020, Doheny et al., 2019, Lehnert et al., 2011). However, our results showed that half of the study group had high healthcare utilisation (≥6), though this was not sensitive for identifying increased likelihood of social care and services. It was surprising that neither multimorbidity nor healthcare utilisation was associated with increased provision of the four most frequently utilised forms of social care and services. Future studies need to explore the role of these frailty factors in relation to social care and services before a reliable conclusion can be drawn from this result.

A systematic review of care for older people with intellectual disabilities based on 42 studies from eight countries found that (i) there was a lack of appropriate services and accommodation, (ii) staff lacked the requisite specialist knowledge, and (iii) retirement for this group meant losing access to services, particularly day services (Innes et al., 2012). These aspects constitute a structure for the discussion that follows.

Concerning the first issue (i), the question of what service and accommodation best meet the needs of older people with intellectual disabilities has been under debate (Bigby, 2002), more recently in Sweden. Our results show that 84% of those with LSS services in the age group 80 years or older lived in *Intellectual Disability residential care*, while 21% of those with SoL care and services in the same age group lived instead in *Elderly residential care* (designed for older people in general). Staff in the disability service have a negative or at best ambivalent attitude to placing people with intellectual disabilities in elderly residential care with staff who are not trained to meet their special needs and who have no experience of communicating with them (Alftberg et al., 2019; Bigby, 2002). Also, older people with intellectual disabilities are a small minority among those in elderly care (Bigby, 2002). The dilemma, though, is that older people with intellectual disabilities are also a minority within the disability service because of the small size of the *Intellectual Disability residential care* units (group homes for 3–5 persons), or because they are living in single-person households. Bigby (2002) argues that neither sector adequately addresses the needs of older people with intellectual disabilities. A few *Intellectual Disability residential care* initiatives in Sweden have started or are in the planning phase within the disability service, with the care staff being trained about ageing. Another alternative is to take the initiative for a model of integrated or coordinated support and care for older people with intellectual disabilities. A recent study on staff perceptions of individual coordinated planning for older people with intellectual disabilities who have comprehensive support and care needs showed that only about half of the older people and the next of kin were present at these meetings (Ahlström et al., 2020). Further research is called for to understand the barriers underlying this low frequency of participation.

On the second issue (ii), the systematic review on ageing among people with intellectual disabilities found that staff lacked knowledge and methods relevant to ageing issues for this group, and concluded that this is why these people did not receive the support they needed in the current welfare system (Innes et al., 2012). Our results confirm this in showing that the oldest age group received the lowest number of LSS social care services, which can be set against the fact that a large number of publications have shown that older people with intellectual disabilities have worse health, polypharmacy and a greater amount of acute (unplanned) inpatient care than people of the same age in the population without intellectual disabilities (Axmon et al., 2017; Coppus, 2013; Hermans and Evenhuis, 2014; McCarron et al., 2013; O’Dwyer et al., 2016; Sandberg et al., 2016). Qualitative studies from Sweden have shown that old age was not addressed in *Intellectual Disability residential care* and care staff felt unprepared to meet age-related changes in older people with intellectual disabilities (Alftberg et al., 2019; Johansson et al., 2017; Kåhlin et al., 2016). There is a clear risk that these changing needs will be overlooked because of behavioural disorders and communication difficulties, which is to say that care staff without sufficient knowledge of ageing will misinterpret health problems as a sign of intellectual disabilities (Bond et al., 2019; Mason and Scior, 2004; O’Dwyer et al., 2018). Thus, the quality of life of older people with intellectual disabilities is dependent on members of the care staff identifying their particular needs. Failure in this respect may lead to worsening of health, more healthcare visits, polypharmacy and the side effects of inappropriate medication. The WHO healthy ageing policy is an initiative committed to developing and maintaining a long, healthy life to support well-being in old age for all citizens (World Health Organization, 2001), which include also older people with intellectual disabilities. Therefore, with the increased number and greater life expectancy of older people with intellectual disabilities, the staff working in social care services need to understand ageing in intellectual disability and implement preventive health interventions. Despite this, the Swedish disability service has no specific guidelines or strategic plan for social care services for older people with intellectual disabilities (Ahlström et al., 2020). This indicates that managers in the disability service need to initiate the development of guidelines and staff training about ageing and preventive interventions.

The third conclusion (iii) of the systematic review on ageing among people with intellectual disabilities was that when people with intellectual disabilities retire, they lose access to social care services, particularly daily activities. These activities are intended for people of working age who are unemployed and do not study (SFS2020:441); thus, no person aged 67 years or older is entitled to receive this service, but the legal situation was not clarified until after our study (Supreme Administrative Court, 2014). This explains our result with rather a high number of retired persons with daily activities. If our research was to be carried out using more recent data, the results would show fewer persons with *Daily activities*, which should be borne in mind when interpreting our findings. People with intellectual disabilities retire under different conditions than the general population. Few have had paid employment during their adult years, and their social network has been limited, often without a spouse or children. Few have accumulated savings for use after their retirement, and most have always been dependent on income support and support from social service for managing everyday living (Bigby, 2002). Bigby and Knox (2009) found that older people with intellectual disabilities wanted to remain active and connected with other people but being retired reduced their ability to participate in previous activities, and their network was even more diminished. This problem has also been raised by care staff in Sweden (Alftberg et al., 2019). Despite increased knowledge of this ageing population, no preparation programmes for facilitating this life change have been reported (Innes et al., 2012).

### Method discussion

This study has strengths and weaknesses, and the results should be interpreted with these in mind. Its strengths are the identification of older people with intellectual disabilities on a national level, which increases the generalisability of the results to similar contexts. The longitudinal register data is based on mandatory registration, and the responsible registrar is the National Board of Health and Welfare, a Government authority. This guarantees continuous quality assurance of data. Also, the NPR register for specialist healthcare has been validated in several studies and has demonstrated high validity and sensitivity (Ludvigsson et al., 2011; Rück et al., 2015).

One weakness is that *Level of Intellectual Disability* was only identified for 14.5% of the study population, which needs to be taken into account when assessing the reliability of this result. The person’s level of intellectual disability is determined early in life after a thorough examination, often in a paediatric clinic. As mentioned previously, the diagnoses were selected from the NPR register, which is based on main cause or contributory cause for received specialist health care. Our study shows that the patient’s level of intellectual disability was not recorded in the majority of cases when the physician treated older people with intellectual disabilities within specialist healthcare. The next weakness is the proportion of people with *Specialist healthcare* visits (50%) and *Multimorbidity* (85%). Despite these high proportions, both of these frailty factors are underestimates because the NPR does not include any data from primary healthcare. In Sweden, primary healthcare is administered at the regional level and the 21 regions started their own registers at different times during recent decades. The current absence of national primary health care data needs to be taken into account. A similar underestimation can also be assumed for *Polypharmacy*, defined in terms of specific drugs that were assessed as being of importance for common sicknesses among older people with and without intellectual disabilities; we expect that other medications besides these were prescribed for the study group. Despite these underestimations, our results show how vulnerable this group is. Another weakness is the lack of information on the provision of *Personal assistance* for more than 20 hours a week. This data is only available in a register within the social security agency, not in the LSS register. However, a low degree of personal assistance is expected because 76% of our study group lived in *Intellectual Disability residential care* with staff available 24 hours per day, and *Home help* was shown to increase with age for those not living at *Intellectual Disability -* or *Elderly residential care*. The services *Intellectual Disability residential care, Elderly residential care* and *Home help* cannot be combined with *Personal assistance*. A further potential threat to the validity of the results is that people without LSS support are not included in the study; however, considering the age group studied, we estimate that the number of people with parents alive and in good enough health to take care of an older child with intellectual disability, without any help from the municipality, is diminishingly small.

Finally, in this study, we used the term intellectual disability according to the criteria for providing social care and services, including autism or autism spectrum disorder (ASD). In Swedish clinical practice, autism spectrum disorder was rarely diagnosed before 1990 (Hirvikoski et al., 2016), which means that the great majority in this older study cohort have intellectual disabilities. We identified people with ASD in a previous study in this project, corresponding to 7.6% of this study cohort (n = 7936) (Nylander et al., 2018). About half of them (57%, 345 persons) had information about intellectual disability diagnosis. This limited assessment of ASD among the older people with intellectual disabilities in Sweden is a weakness, which needs to be considered when interpreting the generalisability of the result.

## Conclusion

To the best of our knowledge, this is the first research study that investigates the association between frailty factors and the provision of community-based social care and services for older people with intellectual disabilities. The services *Intellectual Disability residential care*, *Daily activities*, *Contact person*, *Home help* and *Security alarm* were the most common in all age groups. Age, polypharmacy and severe levels of intellectual disability were the frailty factors associated with significantly increased social care and services. Those in the age group 65–79 years with *polypharmacy* and *moderate/severe/profound* intellectual disabilities were most likely to live in *Intellectual Disability residential care*. However, among those aged 80 years or older, our data revealed that the provision of social care and services was limited compared to the younger age groups. Future research needs to focus on how frailty factors and the ageing process receive attention in social care and service and when there is longstanding medication, especially for persons with severe intellectual disabilities who have difficulty communicating their health problems. Furthermore, there is a need to develop a national strategic plan for disability services with regard to ageing and preventive interventions in order to ensure the best possible healthy ageing for older people with intellectual disabilities.
